# Overexpression of miR-135b-5p promotes unfavorable clinical characteristics and poor prognosis via the repression of SFRP4 in pancreatic cancer

**DOI:** 10.18632/oncotarget.19150

**Published:** 2017-07-10

**Authors:** Xu Han, Hexige Saiyin, Junjie Zhao, Yuan Fang, Yefei Rong, Chenye Shi, Wenhui Lou, Tiantao Kuang

**Affiliations:** ^1^ Department of General Surgery, Zhongshan Hospital, Fudan University, Shanghai, China; ^2^ The State Key Laboratory of Genetic Engineering, School of Life Sciences, Fudan University, Shanghai, China

**Keywords:** miR-135b-5p, SFRP4, clinical characteristics, prognosis, tumorigenesis

## Abstract

Pancreatic ductal adenocarcinoma (PDAC) is a highly aggressive and malignant neoplasm. The aberrant expression of miR-135b-5p and secreted frizzled-related protein 4 (SFRP4) has been revealed to be involved in various cancers. However, the clinical significance of miR-135b-5p and that of its potential target SFRP4 in PDAC remain to be elucidated. Here, we found that miR-135b-5p was markedly upregulated in pancreatic cancer tissue compared with corresponding adjacent normal tissue, whereas SFRP4 was significantly downregulated. The expression of miR-135b-5p was negatively correlated with the expression of SFRP4. PDAC patients with regional lymph node metastases, vascular invasion, tumor microthrombus and higher PET-CT SUVmax values had significantly higher expression of miR-135b-5p. Immunoblotting revealed that regional lymph node metastases were correlated with expressive states of SFRP4. Negative SFRP4 expression was significantly associated with old age, larger tumor size, regional lymph node metastasis and poor differentiation. Survival analyses demonstrated that miR-135b-5p and SFRP4 could predict outcomes and that miR-135b-5p was an independent predictor. *In vitro*, the overexpression of miR-135b-5p promoted the migration and proliferation of PANC-1 and MiaPaCa-2 cells, while immunoblotting demonstrated the downregulation of SFRP4 and the upregulation of beta-catenin. Inhibition of miR-135b-5p suppressed migration, induced apoptosis of PANC-1 and AsPC-1 cells, and reduced the expression of beta-catenin. A luciferase reporter assay confirmed that miR-135b-5p repressed the expression of SFRP4 via the direct targeting of its 3’-untranslated regions. In conclusion, the overexpression of miR-135b-5p and the downregulation of SFRP4 were associated with unfavorable clinical characteristics and poor prognosis, and SFRP4 was shown to be a direct downstream target of miR-135b-5p. Thus, the mechanism that underlies the miR-135b-5p-SFRP4-Wnt/beta-catenin axis represents a potential target for PDAC diagnosis and therapy.

## INTRODUCTION

Pancreatic cancer, which is generally referred to as pancreatic ductal adenocarcinoma (PDAC), is a highly aggressive and nearly lethal neoplasm among the most intractable of human malignancies with a 5-year overall survival rate of less than 10% [1, 2]. Although numerous studies have verified that multiple signaling pathways are involved in the development and metastasis of this cancer type, the molecular mechanisms that regulate the tumorigenesis and metastatic cascades of pancreatic tumors are complex, and our current knowledge regarding these mechanisms is scarce.

MicroRNAs (miRNAs) are noncoding short single-stranded RNA molecules that are known to regulate post-transcriptional gene expression by binding to complementary sequences in the 3′ untranslated region (UTR) of their target mRNAs; this binding consequently leads to mRNA translational repression or degradation [3]. Aberrant miRNA expression is involved in the occurrence and development of various malignant tumors. Specific miRNA profiles in pancreatic cancer tissues have been reported and are distinguished from those of chronic pancreatitis tissues, normal pancreas, and even ampullary adenocarcinoma tissues [4, 5]. Moreover, miRNA expression profiles in pancreatic cancer could be utilized for early diagnosis, molecular subtyping and stratification, clinical behavior and survival prediction [6]. Several studies have reported that miR-135b-5p is aberrantly expressed in malignant neoplasms and that this miRNA participates in several biological processes [7, 8, 9]. However, the functions and mechanisms by which miR-135b-5p is involved in pancreatic cancer remain to be elucidated.

SFRP4 is the gene that encodes secreted frizzled-related protein 4, which is the largest member of the SFRP family of glycoproteins; this protein binds Wnt ligands as an antagonist and inhibits the canonical Wnt signaling pathway [10, 11]. The Wnt pathway is involved in numerous cellular processes and biological functions, and has been suggested to be a regulator of tumorigenesis that generally acts to increase cell proliferation and decrease apoptosis [12]. The canonical Wnt signaling pathway is dependent on β-catenin, and has been investigated in two different states: an inactive ‘off’ state that occurs as a result of Wnt inhibition by antagonists such as SFRPs, and an active ‘on’ state where Wnt signaling is sensitized and initiated by Wnt ligands that bind to frizzled receptors (Fzd) and the co-receptor LRP5/6 [13]. Mounting evidence has suggested that, as a soluble Wnt inhibitor, SFRP4 may act as a tumor suppressor. SFRP4 has also been shown to inhibit proliferation and metastatic potential in malignant neoplasms [14, 15]. Promoter hypermethylation and downregulation of SFRP4 has been illustrated in various cancers [16, 17]. However, according to some studies, conflicting evidence exists in the overexpression patterns of SFRP4 in colorectal carcinomas compared with adjacent normal tissues [18, 19]. Neither the role of SFRP4 as an oncogene or tumor suppressor nor its role in the carcinogenesis, progression and outcome of pancreatic cancer has been confirmed.

The publicly available bioinformatic algorithms analysis showed that SFRP4 might be an important target gene of miR-135b-5p, but this has not yet been confirmed. In this study, we investigated microRNA and protein expression profiles of miR-135b-5p and SFRP4 using samples from patients with pancreatic cancer. We also explored the relationship between the expression profiles of miR-135b-5p and SFRP4 and the clinicopathological features and prognoses of patients with pancreatic cancer. We further experimentally confirmed that miR-135b-5p directly and functionally targeted and suppressed SFRP4, which is a key component of canonical Wnt signaling.

## RESULTS

### miR-135b-5p was upregulated in pancreatic cancer specimens

**Figure 1:**
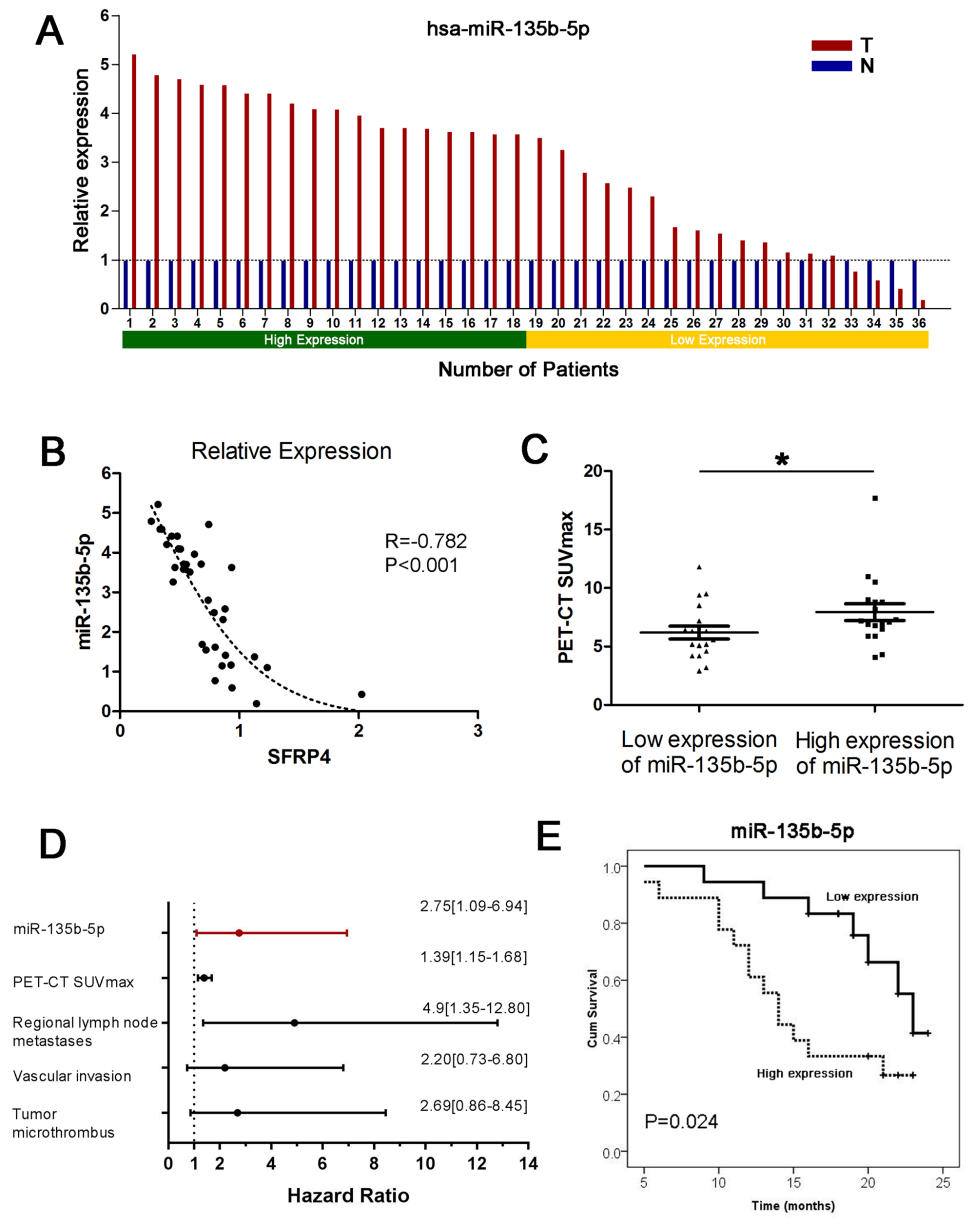
Upregulation of miR-135b-5p in PDAC and its clinical implications **(A)** Expression of miR-135b-5p in 36 cases of fresh human PDAC tissues and matched adjacent normal tissues by real-time PCR analyses. Patient serial numbers were ranked according to the relative expression of miR-135b-5p, and those ranked in the top 50% in terms of expression level were considered to have high expression. **(B)** The level of miR-135b-5p correlated with the level of SFRP4 mRNA; an exponential regression was observed between them. **(C)** The level of overexpressed miR-135b-5p was related to higher PET-CT SUVmax values (Mann-Whitney U test, mean ± SEM; *P < 0.05). **(D)** Forest plot of miR-135b-5p, PET-CT SUVmax values, regional lymph node metastases, vascular invasion and tumor microthrombus as independent predictors of overall survival in patients with resectable PDAC. **(E)** Overexpression of miR-135b-5p correlated with overall survival in patients with resectable PDAC according to univariate analyses.

The miR-135b-5p expression data were compared between pancreatic cancer tissues and corresponding adjacent normal pancreas in 36 paired primary PDACs using qRT-PCR. We found that miR-135b-5p was remarkably upregulated in pancreatic cancer tissue compared with corresponding adjacent normal tissue, as shown in Figure 1A (P<0.001, paired sample t test). An increase in miR-135b-5p expression was found in 88.9% (32/36) of PDAC patients. In addition, a Pearson correlation analysis verified that miRNA expression of miR-135b-5p was negatively correlated with mRNA expression of SFRP4. Moreover, the regression analysis showed a nonlinear exponential regression between SFRP4 (X) and miR-135b-5p (Y) expression (R=-0.782, P<0.001, Figure 1B).

### Correlation of miR-135b-5p expression levels with clinicopathological characteristics and prognosis

In order to further evaluate the clinical significance of miR-135b-5p expression in patients with pancreatic cancer, we categorized miR-135b-5p expression levels in a cohort of 36 PDAC patients into a high expression group and a low expression group, as previously mentioned. We found that patients with regional lymph node metastases, vascular invasion and tumor microthrombus had significantly higher expression levels of miR-135b-5p (P<0.05, Table 1). Interestingly, patients with higher expression levels of miR-135b-5p also had higher PET-CT SUVmax values (P<0.05, Table 1, Figure 1C). A survival analysis indicated that patients with high miR-135b-5p expression had a significantly dismal prognosis compared with those with low miR-135b-5p expression (P=0.024, Figure 1E). Multivariate analyses illustrated that only miR-135b-5p (HR=2.75, 95%CI=1.09-6.94, Figure 1D), PET-CT SUVmax values, regional lymph node metastases, vascular invasion and tumor microthrombus were independent predictors (P<0.05) of OS.

**Table 1:** Associations between expression levels of miR-135b-5p and clinicopathological characteristics at baseline

Characteristics	High expression of miR-135b-5p (n=18)	Low expression of miR-135b-5p (n=18)	P value
Age at onset, mean (SD) (median), y	67.5 (5.5) (67.5)	68.2 (10.1) (71.0)	0.424
Male, n (%)	7 (36.8%)	12 (63.2%)	0.095
Female, n (%)	11 (64.7%)	6 (35.3%)	0.339
Tumor size, mean (median), cm	3.6 (3.3)	3.2 (3.0)	
CA 19-9 level, mean (SD) U/mL	372.9 (612)	181.1 (223)	0.938
CEA level, mean (SD) ng/mL	4.4 (3.8)	6.8 (12.1)	0.563
Regional lymph node metastases, n (%)	16 (88.9%)	9 (50.0%)	0.011*
PET-CT SUVmax, mean (SD)	7.9 (3.0)	6.2 (2.3)	0.034*
Location, n (%)			0.180
Head	6 (37.5%)	10 (62.5%)	
Distal	12 (60.0%)	8 (40.0%)	
Extrapancreatic organ invasion, n (%)	10 (55.6%)	7 (38.9%)	0.317
Neural invasion, n (%)	16 (88.9%)	16 (88.9%)	0.596
Vascular invasion, n (%)	5 (27.8%)	0 (0%)	0.016*
Tumor microthrombus, n (%)	5 (27.8%)	0 (0%)	0.016*
Necrosis, n (%)	2 (11.1%)	1 (5.6%)	0.546
Differentiation, n (%)			0.717
Grade 2	5 (45.5%)	6 (54.5%)	
Grade 2-3	13 (52.0%)	12 (48.0%)	
Ki-67 index, mean (SD) (%)	42.2 (20.2)	30.6 (15.9)	0.079

*P < 0.05

### SFRP4 was suppressed in pancreatic cancer specimens

**Figure 2:**
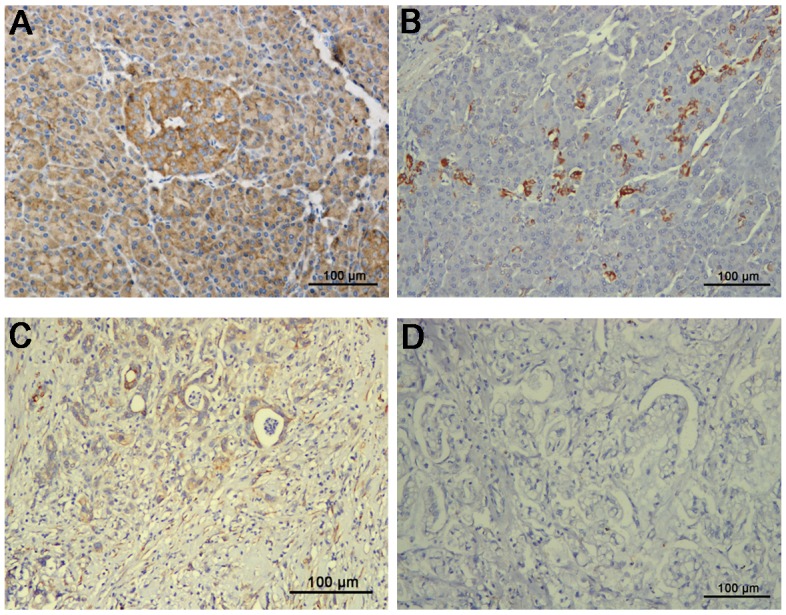
Downregulation of SFRP4 in PDAC and its clinical implications **(A)** The relative expression of SFRP4 mRNA in 36 cases of fresh human PDAC tissues and matched adjacent normal tissues in the same serial section was compared. **(B)** Western blotting indicated that patients without regional lymph node metastases (LN-) had relatively high expression of SFRP4. **(C)** The downregulation of SFRP4 was correlated with poor overall survival in patients with resectable PDAC according to univariate analyses.

As mentioned above, the expression of SFRP4 mRNA was also compared between pancreatic cancer tissues and corresponding adjacent normal pancreas in 36 paired primary PDACs using qRT-PCR. We discovered that SFRP4 was significantly downregulated in pancreatic cancer tissue compared with corresponding adjacent normal tissue, as shown in Figure 2A (P<0.001, paired sample t test). The decrease in SFRP4 was found in 88.9% (32/36) of patients with PDAC.

**Figure 3:**
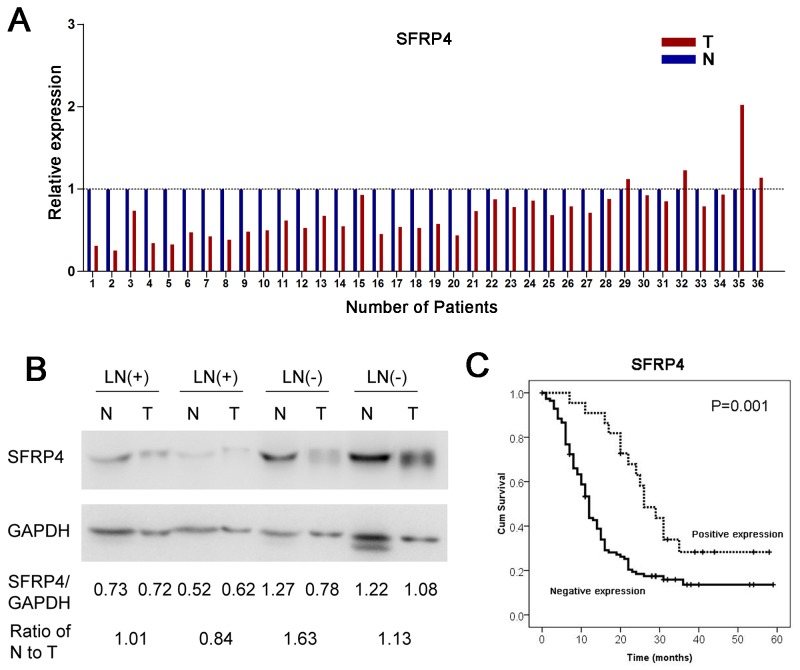
Immunohistochemical expression of SFRP4 protein in cancer tissues and matched adjacent normal tissues from 200 patients with resectable PDAC showed cytoplasmic staining **(A)** SFRP4 was predominantly strongly positive in normal ductal epithelial cells. **(B)** Normal ductal epithelial cells were weakly positive for SFRP4 in only a small portion of patients. **(C)** SFRP4 was weakly positive in PDAC tissues in only a small portion of patients. **(D)** SFRP4 expression was predominantly absent in PDAC tissues.

In another cohort, samples from 200 patients with PDAC were subjected to immunohistochemistry for SFRP4 expression. Adjacent normal pancreas exhibited weak to strong SFRP4 expression in normal ductal epithelial cells when cytoplasmic staining was observed (Figure 3A, 3B). In contrast, in the pancreatic cancer tissues, SFRP4 was predominantly negatively expressed in normal ductal epithelial cells, but a small number of cells exhibited weak positivity (Figure 3C, 3D). The percentages of samples that were negative or weakly positive for SFRP4 expression were 87.5% and 12.5%, respectively.

### Correlation of SFRP4 expression with clinicopathological characteristics and prognosis

According to SFRP4 expression, we categorized SFRP4 protein expression patterns in a cohort of 200 PDAC patients into a negative expression group and a positive expression group, as previously mentioned. The absence of SFRP4 expression was significantly associated with old age, larger tumor size, regional lymph node metastases and poor differentiation (P<0.05, Table 2). A univariate analysis showed that patients with SFRP4 expression had a remarkably favorable prognosis compared with those with negative SFRP4 expression (P=0.001, HR=0.42, 95%CI=0.24-0.73, Figure 2C).

**Table 2:** Associations between the expression of SFRP4 and clinicopathological characteristics at baseline

Characteristics	Positive expression of SFRP4 (n=25)	Negative expression of SFRP4 (n=175)	P value
Age at onset, mean (SD) (median), y	56.0 (10.1) (55.0)	61.0 (10.1) (61.0)	0.021*
Male, n (%)	13 (12.0%)	95 (88.0%)	0.830
Female, n (%)	12 (13.0%)	80 (87.0%)	0.018*
Tumor size, mean (median), cm	3.1 (2.8)	3.5 (3.2)	
Regional lymph node metastases, n (%)	5 (25.0%)	76 (52.1%)	0.023*
Location, n (%)			0.359
Head	19 (76.0%)	6 (24.0%)	
Distal	117 (66.9%)	58 (33.1%)	
Extrapancreatic organ invasion, n (%)	22 (88.0%)	166 (94.5%)	0.177
Neural invasion, n (%)	16 (64.0%)	134 (76.6%)	0.175
Vascular invasion, n (%)	4 (16.0%)	19 (10.9%)	0.451
Tumor microthrombus, n (%)	3 (12.0%)	17 (9.7%)	0.722
Necrosis, n (%)	2 (8.0%)	11 (6.3%)	0.745
Differentiation, n (%)			0.022*
Grade 1	5 (29.4%)	12 (70.6%)	
Grade 2	8 (10.3%)	70 (89.7%)	
Grade 2-3	8 (20.5%)	31 (79.5%)	
Grade 3	4 (6.1%)	62 (93.9%)	

*P < 0.05

Similarly, western blotting revealed that, compared with patients with regional lymph node metastases (LN+), patients without regional lymph node metastases (LN-) had a high SFRP4 expression ratio of normal tissue to tumor tissue (Figure 2B). The findings reported here are consistent with our previous results for SFRP4 expression, in which SFRP4 expression was correlated with regional lymph node metastases.

### miR-135b-5p directly targeted SFRP4 and regulated the Wnt/beta-catenin pathway

**Figure 4:**
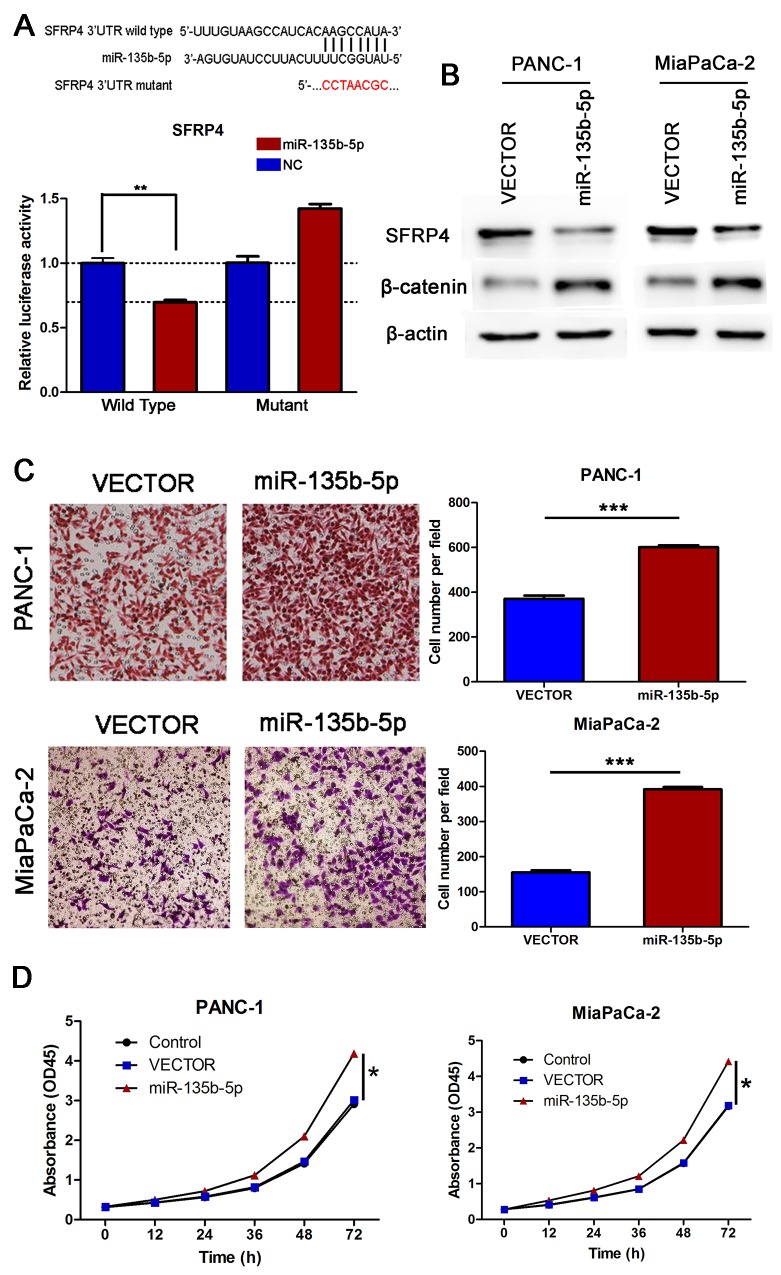
SFRP4 was a direct downstream target of miR-135b-5p. Overexpression of miR-135b-5p promoted migration and proliferation *in vitro* **(A)** Top, miR-135b-5p and its predicted binding sequence in the 3′ UTR of SFRP4. A mutant miR-135b-5p binding sequence was generated in the matched seed region. Bottom, the evaluation of luciferase activity. Co-transfection of HEK-293T cells with a wildtype or a mutant SFRP4 3′UTR with miR-135b-5p mimics. Firefly luciferase activity was examined and standardized to Renilla luciferase activity (Student’s t test, mean ± SEM; **P < 0.01). **(B)** Detection of SFRP4 downregulation and beta-catenin upregulation due to miR-135b-5p overexpression in PANC-1 and MiaPaCa-2 cells by immunoblotting. **(C)** The migratory properties of PANC-1 and MiaPaCa-2 cells transfected with Vector or miR-135b-5p mimics were analyzed using Boyden chambers (Student’s t test, mean ± SEM; ***P < 0.001). **(D)** Proliferation of PANC-1/miR-135b-5p and MiaPaCa-2/miR-135b-5p cells was analyzed by CCK-8 assay at different time points (one-way ANOVA, mean ± SEM; *P < 0.05).

**Figure 5:**
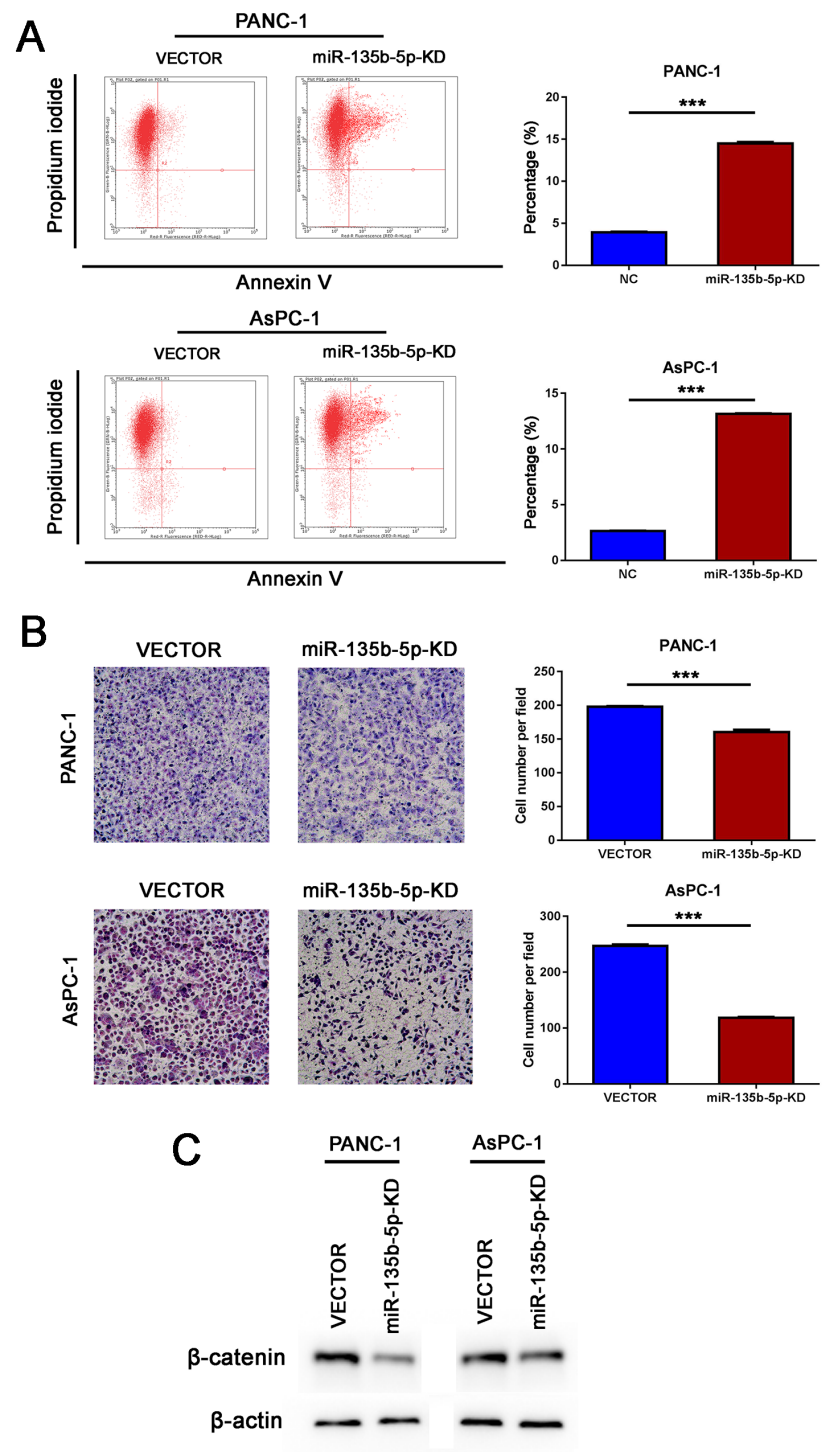
Inhibition of miR-135b-5p suppressed migration and induced apoptosis *in vitro* **(A)** Knockdown of miR-135b-5p may induce late apoptosis of PANC-1 and AsPC-1 cells, as detected by flow cytometry (Student’s t test, mean ± SEM; ***P < 0.001). **(B)** The migratory properties of PANC-1 and AsPC-1 cells transfected with Vector and those of miR-135b-5p-KD cells were analyzed using Boyden chambers (Student’s t test, mean ± SEM; ***P < 0.001). **(C)** Detection of beta-catenin downregulation due to miR-135b-5p repression in PANC-1 and AsPC-1 cells by immunoblotting.

As Wnt signaling pathways are the major molecular pathways that promote tumor progression, we hypothesized that miR-135b-5p might regulate Wnt signaling. Interestingly, using the publicly available algorithms TargetScan and miRanda, we found evidence that miR-135b-5p might directly target SFRP4. The wild type and mutant 3′ UTRs of SFRP4 were cloned and inserted into a luciferase reporter vector. We exogenously overexpressed miR-135b-5p via the transfection of cells with a miR-135b-5p mimic. The luciferase activity of the wild type 3′UTR of SFRP4 was downregulated in the presence of miR-135b-5p (P=0.0023), while the expression of miR-135b-5p did not repress luciferase activity driven by the mutant 3′UTR of the miR-135b-5p-binding seed region (Figure 4A). Moreover, western blotting analysis exhibited that miR-135b-5p overexpression significantly suppressed SFRP4 protein expression levels (Figure 4B). Furthermore, we demonstrated that the overexpression of miR-135b-5p induced the upregulation of beta-catenin (Figure 4B), whereas the inhibition of miR-135b-5p reduced the expression of beta-catenin (Figure 5C). Hence, our results indicate that SFRP4 was a direct downstream target of miR-135b-5p and that miR-135b-5p-SFRP4 might regulate the Wnt/beta-catenin pathway.

### Upregulation of miR-135b-5p promoted migration and proliferation *in vitro*

To clarify the function of miR-135b-5p in PANC-1 cells, we next investigated the role of miR-135b-5p upregulation in the migratory and proliferative properties of these cells *in vitro*. We found that transfection of PANC-1 and MiaPaCa-2 cells with a miR-135b-5p mimic significantly promoted their migration *in vitro* (Figure 4C). Moreover, we also examined the effect of miR-135b-5p expression on cell growth by CCK-8 assay. As shown in Figure 4D, the proliferation rate of miR-135b-5p-transduced PANC-1 cells was remarkably higher compared with that of the vector control cells. These results indicated that overexpression of miR-135b-5p promoted the migratory and proliferative potential of pancreatic cancer cells.

### Inhibition of miR-135b-5p suppressed migration and induced apoptosis *in vitro*

As shown in Figure 5A, the knockdown of miR-135b-5p could remarkably suppress the migration of PANC-1 and AsPC-1 cells *in vitro*. Flow cytometry analysis demonstrated that the knockdown of miR-135b-5p may have induced apoptosis of PANC-1and AsPC-1 cells (Figure 5B). These findings demonstrated that the inhibition of miR-135b-5p suppressed migration and induced apoptosis.

## DISCUSSION

In general, patients with advanced-stage PDAC have a poor prognosis and a high mortality rate, and it is often difficult to efficiently predict metastatic behavior, recurrence, and prognosis. Recently, mounting evidence has suggested that noncoding RNAs play significant roles in the development and progression of human neoplasms, notably of pancreatic cancer [20]. Some miRNA biomarkers have been investigated and found to be useful predictors, and many articles have reported these miRNA functions and have inferred their value in clinical diagnostic and prognostic applications. miR-135b-5p was previously reported to promote carcinogenesis and tumor development in humans, but few studies have been conducted in patients with pancreatic cancer [21, 22, 23]. miR-135b has been identified as one of the most highly dysregulated miRNAs in a small sample of fresh PDACs [24]. However, further insights into the roles and molecular mechanisms of miR-135b-5p during the pathogenesis of PDAC are needed.To investigate the clinical significance and the precise mechanism of action of miR-135b-5p in the pathogenesis of PDAC, we revealed the miR-135b-5p was significantly overexpressed in PDAC tissues compared with matched noncancerous tissues. The upregulation of miR-135b-5p was remarkably correlated with aggressive clinicopathological features such as regional lymph node metastases, vascular invasion and tumor microthrombus, which suggests that miR-135b-5p might be associated with the progression of PDAC. In addition, tumors in which miR-135b-5p was upregulated also had a higher Ki-67 index, but the P value was very close to the cutoff due to the small sample size. Furthermore, overexpressed miR-135b-5p was associated with higher PET-CT SUVmax values, which were used to semi-quantitatively estimate glucose uptake ability. This result might reflect a link between miR-135b-5p and PDAC metabolism since ^18^FDG-PET is a functional imaging method that allows for the visualization of glucose uptake by tumors *in vivo*. Consistently, increased miR-135b expression was positively correlated with HIF-1α expression and microvessel density in a model of HNSCC, which affected tumor metabolism [25]. More importantly, overexpressed miR-135b-5p was also correlated with poorer overall survival of patients with PDAC, which suggests that this miRNA might be an effective independent predictor of outcome. According to the functional assay, the overexpression of miR-135b-5p promoted the migration and proliferation of pancreatic cancer cells *in vitro*. In addition, the inhibition of miR-135b-5p suppressed migration and led to apoptosis of pancreatic cancer cells *in vitro*. Thus, our results demonstrated that miR-135b-5p might act as an oncogenic miRNA and may be a candidate for targeted molecular therapy in PDAC.

Currently, we know that miRNA molecules regulate gene expression at the post-transcriptional level via either the inhibition of translation initiation or via direct mRNA cleavage. The publicly available bioinformatic databases suggested that SFRP4 might be important target genes of miR-135b-5p. Consequently, we demonstrated that overexpressed miR-135b-5p was negatively correlated with downregulated SFRP4 *in vivo*, which suggested that the downregulation of SFRP4 in PDAC might be caused by an upregulation of miR-135b-5p. Importantly, *in vitro*, the luciferase reporter assay confirmed that SFRP4 was a direct downstream target of miR-135b-5p. Similar results were found when miR-135b was inhibited in mouse models of CRC; this resulted in a reduction of tumor growth since miR-135b controls genes involved in proliferation, invasion, and apoptosis [26]. This evidence suggested that miR-135b-5p directly and effectively targets SFRP4.

Aberrant Wnt signaling has been implicated in tumorigenesis, and inhibition of components of this pathway such as SFRPs, and more specifically, SFRP4, may be a potential target for cancer therapy. Our results revealed that the overexpression of miR-135b-5p induced the upregulation of beta-catenin, whereas the knockdown of miR-135b-5p reduced the expression of beta-catenin. We hypothesized that miR-135b-5p-SFRP4 regulates the Wnt/beta-catenin pathway and affects PDAC carcinogenesis. The existing literature indicated that SFRP4 might increase the chemotherapeutic response and may be a potential drug that is used to destroy cancer cells [27, 28]. In our study, SFRP4 mRNA and protein were remarkably downregulated in PDAC tissues compared with matched noncancerous tissues. Similarly, it was reported that the deficiency in SFRP4 expression was found to be 55% in PDAC samples compared with 18% in adjacent tissue samples obtained from patients during surgery. Moreover, hypermethylation of SFRP4occurred in 60% of PDAC samples, whereas this occurred in only 10% of adjacent tissue samples [29]. Furthermore, we found that aggressive clinicopathological features such as old age, larger tumor size, poor differentiation, and especially regional lymph node metastases were associated with negative SFRP4 protein expression in PDAC. Evidence in our study suggested that abnormalities in SFRP4 were associated with pancreatic carcinogenesis and development of PDAC. Although no statistical significance was found in the multivariate survival analysis, a deficiency in the expression of SFRP4 could decrease survival duration according to the univariate analysis. Therefore, SFRP4 has been shown to be a potentially useful protein biomarker in the prediction of outcome.

Taken together, this study demonstrated the functional and mechanistic relationship of the miR-135b-5p-SFRP4-Wnt/beta-catenin axis, and due to its effect on the proliferation, migration and apoptosis of pancreatic cancer miR-135b-5p and SFRP4 cells, components of this axis may be promising biomarkers for the prediction of clinical behaviors and outcomes in patients with PDAC. However, further functional analyses and clinical studies of larger sample sizes are needed in order to understand the role of the miR-135b-5p-SFRP4-Wnt/beta-catenin axis in the carcinogenesis and pathophysiology of PDAC.

## MATERIALS AND METHODS

### Patients and pancreatic cancer specimens

This study included 2 groups of patients with pancreatic ductal adenocarcinoma who underwent radical surgery with regional lymph node resection in the Department of Pancreatic Surgery, Zhongshan Hospital, Fudan University between 2007 and 2011 (the first group consisted of 200 cases) and between 2014 and 2015 (the second group consisted of 36 cases). None of these patients received any preoperative treatment. Institutional Review Board approval and informed consent were obtained for this study. Clinical data were obtained from the Pancreatic Tumor Registry and were analyzed retrospectively. The recorded clinicopathologic characteristics included age, gender, tumor size, CA 19-9 level, CEA level, regional lymph node metastases, PET-CT SUVmax, location, extrapancreatic organ invasion, neural invasion, vascular invasion, presence of tumor microthrombus, necrosis, differentiation, and Ki-67 index. Follow-up information was collected from the Pancreatic Tumor Registry at Zhongshan Hospital, Fudan University. The duration of overall survival (OS) was calculated from the date of surgery until death from PDAC or the patient’s last follow-up.

### RNA extraction and real-time PCR

We investigated miRNA and mRNA expression profiles in 36 pairs of primary PDAC specimens and corresponding adjacent normal pancreatic tissues. In these experiments, total RNA from cells or tissues was extracted using TRIzol reagent (Invitrogen). The expression of miRNAs was verified by qRT-PCR using a microRNA qRT-PCR SYBRGreen Detection Kit (BioTNT Biotech, China) according to the manufacturer’s instructions. The relative expression of mRNAs was examined by qRT-PCR using a SYBRGreen Detection Kit (BioTNT Biotech, China) according to the manufacturer’s protocol. All of these reactions were performed in triplicate in order to reduce bias. We designed reverse transcription stem-loop primers and specific amplification primers. The target sequence of miR-135b-5p was: UAUGGCUUUUCAUUCCUAUGUGA. The mRNA primers were as follows: SFRP4 forward, 5’-GGAGGATGTTAAGTGGATAGA-3’, SFRP4 reverse, 5’-GGAGGATGTTAAGTGGATAGA-3’; β-actin forward, 5’-AAGGTGACAGCAGTCGGTT-3’, β-actin reverse, 5’-TGTGTGGACTTGGGAGAGG-3’. All samples were normalized to endogenous controls and the fold changes in expression were calculated relative to the controls. The relative levels of miRNAs were normalized to the level of the internal control U6 snRNA, while mRNAs were normalized to the level of β-actin. These relative expression levels were calculated using the 2^-ΔΔCT^ method. We defined the patients who ranked in the top 50% in terms of the expression level of miR-135b-5p as the high expression group, while all others were in the low expression group.

### Cell culture

HEK-293T cells and the pancreatic cancer cell lines PANC-1, MiaPaCa-2, and AsPC-1 cells were purchased from The Cell Bank of Type Culture Collection of the Chinese Academy of Sciences (CAS, Shanghai). All cells were cultured in DMEM supplemented with 10% fetal bovine serum (FBS) (Gibco, USA),100 U/ml penicillin, and 100 μg/ml streptomycin. The cells were maintained in a 5% CO_2_ incubator at 37°C.

### Plasmid construction and transfection

The 3′UTR segments of human SFRP4 that were predicted to interact with miR-135b-5p were amplified by PCR and inserted into a pGL3 luciferase reporter plasmid (Promega, USA). We purchased the miR-135b-5p mimic from GeneChem (Shanghai, China). All transient transfections were performed using Lipofectamine 2000 (Invitrogen) according to the manufacturer’s instructions.

### Lentiviral vectors and transfection

Lentiviral vectors to inhibit human miR-135b-5p were constructed by GeneChem (Shanghai, China). Both a recombinant lentivirus (KD) and a negative control lentivirus (GFP-lentivirus) were prepared. Approximately 72h after viral infection, GFP expression was confirmed by fluorescence microscopy. The clones were designated “miR-135b-5p-KD cells”.

### Luciferase reporter system

HEK-293T cells cultured in a 96-well plate were co-transfected with 0.4 μg of miR-135b-5p mimics, 0.1 μg of the firefly luciferase reporter vector containing the wildtype or mutant 3′UTR of SFRP4 and 0.02 μg of the control vector containing Renilla luciferase. Transfections were performed in triplicate and were repeated in three independent experiments. Forty-eight hours after transfection, luciferase activity was analyzed using a Dual-Luciferase Reporter Assay System (Promega, USA) according to the manufacturer’s protocol. Firefly luciferase values were normalized to Renilla luciferase in order to control for transfection efficiency.

### Migration assay

Cell migration assays were performed using 6.5-mm Boyden chambers (8-μm pore size, BD). Briefly, cells (2×10^5^) in culture medium containing 1% FBS were seeded in the upper chamber, and culture medium with 10% FBS was added to the lower chamber as a chemoattractant. Then, 12 to 16 hours later, the cells that had migrated to the basal side of the membrane were fixed and stained, visualized and imaged. Five random fields (magnification×100) were observed for cell counting.

### Cell proliferation assays

After transfection with a miR-135b-5p mimic for 48 h, PANC-1 cells were seeded in 96-well plates at a density of 2,000 cells per well. The culture medium was removed, and aliquots (10 μl) from the CCK-8 kit (Dojindo, Japan) plus 90 μl of complete medium were added to the wells; the plate was then incubated for 2 hours. After incubation, absorbance was determined at 450 nm at different time points (0, 12, 24, 36, 48, 72 h). Each experiment was performed in triplicate.

### Western blotting

Samples from tissues or cell lysates were separated by SDS-polyacrylamide gel electrophoresis, transferred onto polyvinylidene difluoride membranes and then incubated with primary antibodies against the following antigens: SFRP4 (1:1000; Abcam, Cambridge, USA), beta-catenin (1:1000; Abcam, Cambridge, USA) and GAPDH (1:5000; Proteintech, Rosemont, IL, USA). This was followed by an incubation with a horseradish peroxidase (HRP)-conjugated secondary antibody (1:2000; Santa Cruz, USA). Reactive proteins were visualized by an enhanced chemiluminescence system.

### Tissue arrays and immunohistochemistry

To ensure uniform staining conditions of the tumors among all samples, the tissue microarray (TMA) method was selected for 200 samples. The following 2 regions were sampled in each specimen: pancreatic tumor and adjacent normal pancreas. Sections (4 μm in thickness) were cut from formalin-fixed paraffin embedded tissues and placed onto silanized slides. The slides were then stained in a Bond-Max Leica autostainer (Leica Biosystems, Milton Keynes, UK). Antibody detection was performed using the biotin-free Bond Polymer Refine Detection System (DS9800, Leica Microsystems, Newcastle, UK) according to the manufacturer’s protocol. Immunohistochemistry was performed for SFRP4 (1:100; Abcam, Cambridge, MA, USA) in 200 cases. Immunoreactivity was evaluated based on a semiquantitative scale that considers both the extent (score, 0, 1, 2 or 3 for positive cells, <5, 5–40, 40–70, and >70%, respectively) and the intensity (score, 0, 1, 2 or 3 for “−”, “+”, “++”, and “+++”, respectively) of staining. The product was used to obtain a total immunostaining score (range, 0–9). Samples with a score of 0 were considered negative, those with a score of 1 or 2 were considered weakly positive, those with a score of 3 or 4 were considered moderately positive, and those with a score of 6 or 9 were considered strongly positive. For all biomarkers, samples with negative or weakly positive staining were considered to exhibit low expression of a given marker, while those with moderately positive or strongly positive staining were considered to exhibit high expression of a given marker.

### Cell apoptosis analysis

Cell apoptosis was performed by staining with Annexin V-APC (eBioscience, USA) in conjunction with PI according to the manufacturer’s protocol; staining was detected by flow cytometry (Guava easyCyte HT, Millipore).

### Statistical analysis

The statistical analyses were performed with the SPSS statistical package version 16.0 (Chicago, IL). Pearson’s chi-square test and Fisher’s exact test were used to compare proportions when appropriate, whereas means were compared using Student’s t test, non-parametric Mann-Whitney test or one-way ANOVA. Pearson correlation and nonlinear exponential regression analyses were also performed. Univariate OS analyses were performed using the Kaplan-Meier method, and the results were compared by log-rank test. Multivariate analysis using the Cox proportional hazards model was performed to identify the factors that are independently associated with prognosis. Risk factors were described as hazard ratios with 95% confidence intervals (HRs, 95% CI). Statistical significance was defined as P<0.05.
